# Transcranial direct current stimulation (tDCS) in behavioral and food addiction: a systematic review of efficacy, technical, and methodological issues

**DOI:** 10.3389/fnins.2015.00349

**Published:** 2015-10-09

**Authors:** Anne Sauvaget, Benoît Trojak, Samuel Bulteau, Susana Jiménez-Murcia, Fernando Fernández-Aranda, Ines Wolz, José M. Menchón, Sophia Achab, Jean-Marie Vanelle, Marie Grall-Bronnec

**Affiliations:** ^1^Addictology and Liaison Psychiatry Department, Nantes University HospitalNantes, France; ^2^Clinical Investigation Unit 18-BALANCED “BehaviorAL AddictioNs and ComplEx Mood Disorders”, University Hospital of NantesNantes, France; ^3^Department of Psychiatry, University Hospital of Bellvitge-IDIBELLBarcelona, Spain; ^4^Department of Psychiatry and Addictology, University Hospital of DijonDijon, France; ^5^Behavioral Addictions Program, NANT New Addictions New Treatments, Addiction Division, Department of Mental Health and Psychiatry, University Hospital of GenevaGeneva, Switzerland; ^6^CIBER Fisiopatología Obesidad y Nutrición, Instituto de Salud Carlos IIIBarcelona, Spain; ^7^CIBER Salud Mental, Instituto de Salud Carlos IIIBarcelona, Spain; ^8^EA 4275 “Biostatistics, Clinical Research and Subjective Measures in Health Sciences”, University of NantesNantes, France

**Keywords:** transcranial direct current stimulation, neuromodulation, behavioral addiction, craving, eating disorders, food craving, non-invasive brain stimulation

## Abstract

**Objectives:** Behavioral addictions (BA) are complex disorders for which pharmacological and psychotherapeutic treatments have shown their limits. Non-invasive brain stimulation, among which transcranial direct current stimulation (tDCS), has opened up new perspectives in addiction treatment. The purpose of this work is to conduct a critical and systematic review of tDCS efficacy, and of technical and methodological considerations in the field of BA.

**Methods:** A bibliographic search has been conducted on the Medline and ScienceDirect databases until December 2014, based on the following selection criteria: clinical studies on tDCS and BA (namely eating disorders, compulsive buying, Internet addiction, pathological gambling, sexual addiction, sports addiction, video games addiction). Study selection, data analysis, and reporting were conducted according to the PRISMA guidelines.

**Results:** Out of 402 potential articles, seven studies were selected. So far focusing essentially on abnormal eating, these studies suggest that tDCS (right prefrontal anode/left prefrontal cathode) reduces food craving induced by visual stimuli.

**Conclusions:** Despite methodological and technical differences between studies, the results are promising. So far, only few studies of tDCS in BA have been conducted. New research is recommended on the use of tDCS in BA, other than eating disorders.

## Introduction

### Substance use disorder and behavioral addictions

Addictions are complex disorders conventionally represented by substance use disorders (SUDs). Other behaviors without any substance use share many clinical similarities, and are therefore categorized as addictions without drug use,—more commonly called behavioral addictions (BAs) (O'Brien, 2011; Potenza, 2014) -, as evidenced in the recent release of the DSM-5 (American Psychiatric Association, 2013), where gambling disorders now appear in the “substance-related and addictive disorders” category, among other SUDs. Until now, this is the only BA that the task force researchers included into the edited version of the manual. However, for many authors, BAs also encompass video games addiction, Internet addiction, sexual addiction, compulsive buying, sports addiction, and eating disorders (Gearhardt et al., 2011; Farré et al., 2015; Jiménez-Murcia et al., 2015). It has increasingly been suggested that some eating habits, such as the uncontrolled intake of high-calorie food rich in sugar and fat, can also be seen as behavioral addictions and was recently referred to as “food addiction” (Davis and Carter, 2009; Gearhardt et al., 2011; Hebebrand et al., 2014; Schulte et al., 2015).

As in any SUD, one of the key symptoms in BAs is craving, defined as a pressing, urgent, and irrepressible desire to give in to a BA, which results in most cases in a loss of control (Skinner and Aubin, 2010; O'Brien, 2011). The craving contributes to the development, continuation and relapse of an addictive behavior. Although craving is not pathognomonic of addiction, it remains a key symptom in the addictive process, to the point that it is now considered in the DSM-5 as a diagnostic criterion for substance-related and addictive disorders (American Psychiatric Association, 2013). Craving can lead to a loss of control over one's behavior. Executive functions (such as decision making and risk-taking process) and working memory impairments have been found in both SUDs and BAs (Fernández-Serrano et al., 2010; Marazziti et al., 2014). These clinical features suggest that BAs and SUDs may share similar neurophysiopathological abnormalities. Some authors support the idea of common neurochemical and genetic mechanisms involved with both substance and non-substance, addictive behaviors, linked to disturbances of the reward system, so-called “reward deficiency syndrome” (Blum et al., 2014). The central reward pathway involves the dopaminergic system such as the mesolimbic cortical ventral tegmental area and projections to the nucleus accumbens and the prefrontal cortex (Goldstein and Volkow, 2002; García-García et al., 2014). Neuroimanging studies underlined the important function of the prefrontal cortex, especially the dorsolateral prefrontal cortex (DLPFC), in both SUDs and BAs (Goudriaan et al., 2012).

The pharmacological and psychotherapeutic treatments of addictions and of the craving in particular, have shown their limits (Achab and Khazaal, 2011; Marazziti et al., 2014), which indicates the need for new treatment possibilities.

### Non-invasive brain stimulation, a promising treatment for addictions

More recently, new treatment modalities such as non-invasive brain stimulation (NIBS) have been explored in the field of addiction, such as Transcranial Direct Current Stimulation (tDCS) and repetitive transcranial magnetic stimulation (rTMS) (Jansen et al., 2013; Grall-Bronnec and Sauvaget, 2014). rTMS generates a magnetic field in a coil that is placed on the scalp. The magnetic field induces an electrical current in the brain tissue beneath the coil, resulting in alterations of neural excitability (Ziad, 2002). In addition to its cortical action, TMS may act remotely on deeper structures, via brain circuits and interhemispheric connections (Fox et al., 1997). tDCS is another NIBS method capable of modulating cortical excitability (Feil and Zangen, 2010). tDCS consists in delivering a low intensity electric field (1–2 mA) through the brain between two electrodes. The current enters the brain from the anode, travels through the tissue, and exits out the cathode (Higgins and George, 2009). The anodic stimulation increases cortical excitability, whereas the cathodic stimulation reduces it. The administration of tDCS is relatively easy. Electrodes can be placed anywhere on the scalp and are held in place with an elastic headband (Higgins and George, 2009). In general, one session lasts 10–20 min. Two sessions a day can be given easily if required. Like rTMS (Keck et al., 2002; Hanlon et al., 2013), tDCS showed that it could have remote effects (Chib et al., 2013).

rTMS and tDCS, applied to the DLPFC, may transiently modify decision-making, risk-taking, and impulsivity, processes directly linked to behavioral disorders. It has thus been shown that applying tDCS on prefrontal areas modifies the decision process in sane subjects (Fecteau et al., 2007a,b; Knoch et al., 2008; Boggio et al., 2010), but also in addicted subjects (Fecteau et al., 2014). The decision-making process shares common mechanisms with the impulsive behaviors observed in addictions. By modulating it, we could decrease impulsivity in addicted patients, and, indirectly, act on the craving (Fecteau et al., 2010). Anodal tDCS over the DLPFC may enhance executive function and provide improved cognitive control, and thus reduce the probability of relapse to drug use (da Silva et al., 2013).

Finally, even if the neurophysiological effects behind the effects of tDCS on craving are not completely clarified yet, choosing the DLPFC as a stimulation area is justified by the involvement of frontal areas in the neurobiology of eating disorders, either bulimia, or anorexia nervosa (Kaye et al., 2009; van Kuyck et al., 2009; Frank et al., 2013; Friederich et al., 2013). More precisely, the DLPFC might be involved in the food restriction and cognitive control mechanisms, which are linked with the working memory (von Hausswolff-Juhlin et al., 2015).

rTMS and tDCS applied to the DLPFC may therefore indirectly modulate dopaminergic pathways (Addolorato et al., 2012) and may consequently have an impact on the symptoms of addiction (Keck et al., 2002; Feil and Zangen, 2010). Cognitive control could be improved and/or cravings could be reduced (Jansen et al., 2013). So far, tDCS have proven its efficacy to decrease craving, mainly in SUDs (Jansen et al., 2013; Naim-Feil and Zangen, 2013; Kuo et al., 2014). Moreover, reviews and comprehensive work about tDCS in the field of psychiatry and addictions did not have considered BAs (Feil and Zangen, 2010; Kuo et al., 2014; Tortella et al., 2015).

The goal of this study is to conduct a systematic review of the efficacy, and of the technical and methodological stakes of applying tDCS to the field of BAs.

## Methods

This systematic review was conducted and reported in accordance with the Preferred Reporting Items for Systematic reviews and Meta-Analyses (PRISMA) guidelines (Moher et al., 2009).

### Search resources

Two independent reviewers conducted the literature search, including different sources such as electronic databases (PubMed and Science Direct), citations, and reference lists, as well as gray literature. In addition, the reference lists of all included studies were hand searched, limiting the search to articles published in English. To ensure the recency of articles, the search was limited from inception to December, 31st, 2014.

The search terms used were a combination of MESH terms and keywords and included “tDCS” and “addiction,” “anorexia nervosia,” “behavioral addiction,” “bulimia nervosa,” “eating disorders,” “binge eating disorders,” “compulsive buying/shopping,” “craving,” “Dorsolateral prefrontal cortex (DLPFC),” “dependence,” “dopamine,” “eating disorders and not otherwise specified (EDNOS),” “exercise,” “food craving,” “gambling disorder,” “impulsivity,” “Internet addiction,” “pathological gambling,” “risk-taking behavior,” “sex addiction,” and “sports addiction” in the title, abstract, or keywords.

### Eligibility criteria

Studies had to fulfill the following inclusion criteria to be included: the target problem was a BA; the intervention was performed using tDCS; the study was a clinical trial, as defined by the WHO (WHO, 2015)—including randomized controlled trials (RCTs), controlled trials, cohort studies, case-control studies and multiple base-line studies. Exclusion criteria were: clinical studies about tDCS among SUDs; review and didactic articles; physiopathological studies and case reports.

### Study selection

**Figure 1:**
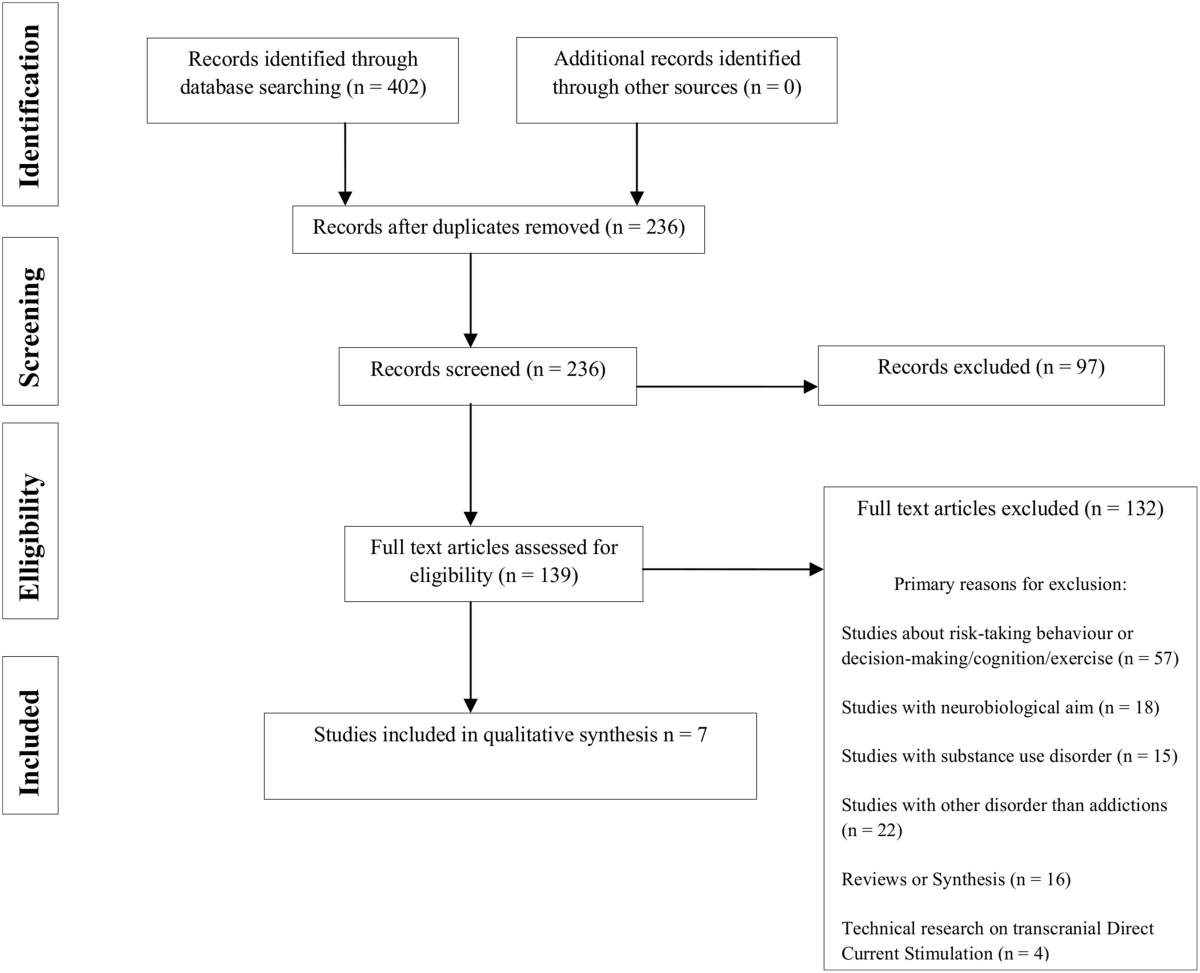
**PRISMA flow chart of literature search**.

First, all studies were screened based on their titles and abstracts. Second, the two reviewers read the full text of all studies identified in this search process. This work was carried out independently using the same bibliographic search. In the event of a disagreement between the two reviewers, the relevant studies were discussed (see Figure 1 for the study selection flow chart).

### Data extraction

Extracted data included clinical, methodological, and technical considerations (see Tables 1, 2).

**Table 1:** **Clinical trials of tDCS and behavioral addictions: general and clinical characteristics**.

**Studies**	**Studied condition**	***N***	**Participants inclusion criteria**	**Mean age (years)**	**Proportion of females**	**Main exclusion criteria**	**Design**	**Objective**	**Main experimental conditions**	**Main outcome measures**	**Main results**	**Drop out (reason)**
Fregni et al., 2008b	Food craving	23	Healthy subjects aged 18–55. Frequent food craving (>3 times/day) and strong urges to eat	23.7	91.3%	NS	Randomized Sham-controlled Double-blind Crossover design	Investigate the effect of tDCS on food cue-induced craving-related behavior	Exposition to food and watching a movie of food associated with strong craving	Food craving (VAS) and food consumption before and after treatment Visual attention to food using an eye tracking system	Craving for viewed foods was reduced by anode right/cathode left tDCS After sham stimulation, exposure to real food or food-related movie increased craving After anode left/cathode right tDCS, the food-related stimuli did not increase craving levels	2 (School work)
Goldman et al., 2011	Food craving	19	Healthy subjects aged 21–70 with frequent food cravings (≥3 times/week during the past month) BMI < 40	32.4	68.4%	Pregnancy History of an ED or depression Suicidality Implanted metal devices History of seizures, brain surgery	Randomized Sham-controlled Single-blind Within-subject crossover design	Investigate the effect of tDCS on food cue-induced craving and the ability to resist foods	Twenty-four images of foods (e.g., ice cream, cheese-burgers, pizza) were presented in random	Food craving and ability to resist tasting (VAS) while viewing food image	Food cravings ratings were reduced in both conditions The percent change in self-reported cravings from pre- to post-stimulation was significantly greater for real stimulation Decrease in food craving, particularly for sweets and carbohydrates No change in food consumption	1 (NS)
Montenegro et al., 2012	Hunger, satiety and desire to eat sensations	9	Overweight subjects 2–3 h fasting	24	44.4%	Cardiovascular disease Pregnancy, History of eating disorders Depression Implanted metal parts	Randomized Single-blinded Sham-Controlled Crossover design	Investigate the effect of tDCS isolated or combined with aerobic exercise on the desire to eat, hunger, and satiety	No exposition to food	Appetite sensations (VAS) evaluated at four moments: baseline; after tDCS; post-exercise; 30 min post-exercise	tDCS on left DLPFC decreased the desire to eat at baseline tDCS associated with exercise had greater suppressing effect in desire to eat compared to either tDCS or exercise alone tDCS associated with exercise decreased hunger and increased satiety immediately after exercise	0
Kekic et al., 2014	Food craving and temporal discounting	17	Healthy women aged 18–60 with frequent food cravings (≥1 per day)	26.4	100%	SUD Major psychiatric disorder Current or past history of an eating disorder Personal or family history of seizures Implanted metal devices Pregnancy	Randomized Double-blind Sham-controlled Within-subjects crossover design	Investigate the effect of tDCS on food cravings, intertemporal choice behavior, actual food consumption and temporal discounting	Exposition to real food	VAS measuring baseline hunger FCT FCQ-S Saliva sample TD task	tDCS reduced cravings for sweet but not savory foods Participants who exhibited more reflective choice behavior were more susceptible to the anticraving effects of tDCS than those who displayed more impulsive choice behavior.	1 (Skin irritation)
Khedr et al., 2014	Urge to restrict food intake	7	Treatment-resistant AN BMI between 14 and 17.5 kg/m2	21.75	85.7%	Drugs (dopaminergic, psychotropic, antiepileptic, or hormonal drugs Estrogen) at least 2 weeks before the study Six patients had been receiving antidepressant (SSRls) which was kept constant throughout the study	Open-label, single-arm study	Evaluate the acceptability of tDCS as a potential treatment for AN Investigate the effect of tDCS on the urge to restrict food intake and symptoms of depression associated with AN.	No exposition to food	EAT EDI BDI	Significant effect of time (pre, post, and 1 month later) on the three rating scores Significant correlation between the percent improvement of BDI and EAT and between BDI and EDI Ten daily sessions of anodal tDCS over the left DLPFC improved symptoms of both depression and AN for up to 1 month	0
Lapenta et al., 2014	Food consumption and ERP-indexed inhibitory control	9	Healthy females. Frequent (>3 times/day) and strong urges to eat	23.4 ± 2 years)	100%	Neuropsychiatric disorder History of abuse of alcohol or another drug Psychiatric medication Pregnancy Eating disorder	Randomized Double-blind Sham-controlled Crossover design	Evaluate the cognitive ERPs that are associated with the effects of DLPFC tDCS on food craving	Exposition to food and watching a movie of food associated with strong craving Go/No-go task that contained pictures of food and furniture (a control visual stimulus).	ERP during a Go/No-Go task Food craving (VAS) while exposed to real food and a movie of food Snack intake; Attentional bias for food (eye tracking)	Active DLPFC tDCS (anode right/cathode left), compared with sham stimulation, reduced the frontal N2 component and enhanced the P3a component of responses to No-go stimuli, regardless of the stimulus condition (food or furniture). Active tDCS was also associated with a reduction in caloric intake	0
Jauch-Chara et al., 2014	Food intake	14	Healthy young normal-weight men with BMI from 20 to 25. Low cognitive restraint, low disinhibition, and normal susceptibility to hunger scores	24.8	0%	Any medication Acute and chronic medical diseases Alcohol or drug abuse, Smoking Participation in competitive sports Disturbances in sleep continuity	Randomized Sham-controlled Single-blind Code-based, Counter-balanced Crossover design	Investigate the effect of repetitive tDCS to the right DLPFC on food intake	Exposition to food and consomption	Subjective appetite (ratings and VAS) Food intake behavior from a standardized *ad libitum* buffet	tDCS reduced food consumption in humans	0

*AN, Anorexia Nervosia; BDI, Beck Depression Inventory; BMI (in kg/m2), Body Mass Index; DLPFC, Dorsolateral Prefrontal Cortex; EAT, Eating Attitude Test; EDI, Eating Disorder Inventory; ERPs, event-related potentials; FCQ-S, Food Craving Questionnaire-State; FCT, Food Challenge Task; NS, Not Specified; SUD, Substance Use Disorders; TD task, temporal discounting task; SSRls, serotonin reuptake inhibitors; tDCS, transcranial direct current stimulation; VAS, Visual Analog Scales*.

**Table 2:** **Clinical trials of tDCS and behavioral addictions: technical characteristics**.

**Studies**	**Procedure of tDCS number of sessions**	**Duration (min/session)**	**Anode**	**Cathode**	**Current density (A/m2)**	**Sham conditions**	**Tolerance/Adverse effects**
Fregni et al., 2008b	Three types of bilateral stimulation of DLPFC (48 h ii)—1 session	–	–	–	–	–	Mild and similar in the three conditions of stimulation Most frequent adverse effects were scalp burning, headache, local itching, burning sensation, and somnolence
	Active anode left/cathode right tDCS	20	F3	F4	2 mA	–	
	Active anode right/cathode left tDCS	20	F4	F3	2 mA	–	
	Sham tDCS	20	–	–	–	Electrodes were placed at the same positions as in active stimulation The stimulator was turned off after 30 s of stimulation	
Goldman et al., 2011	Two types of bilateral stimulation of DLPFC (48–72 h ii)—1 session	–	–	–	–	–	NS
	Active anode right Cathode left tDCS	20	F4	F3	2 mA		
	Sham tDCS with the same electrode placement	20	F4	F3	–	The tDCS device was turned up to 2 mA for 30 s, then slowly ramped-down to 0 mA over the period of 1 min, and finally turned off for the duration of the 20 min session Participants were asked to guess whether they received real or sham stimulation at each session, as well as how confident they were in their guess	
Montenegro et al., 2012	Two types of unilateral stimulation over DLPFC (48–120 h ii)—1 session	–	–	–	–	–	NS
	Anodal unilateral stimulation on left DLPFC, alone or combined with isocaloric exercise bouts	20	F3	Fp2	2 mA		
	Sham tDCS with the same electrode placement, alone, or combined with isocaloric exercise bouts	20	F3	Fp2	–	The stimulator was turned off after 30 s	
Kekic et al., 2014	Two types of bilateral stimulation of DLPFC (≥48 h) ii)—1 session	–	–	–	–	–	One participant withdrew from the study after the first appointment due to skin irritation at the site of stimulation Slight headache following active tDCS Participants reported experiencing minimal Discomfort
	Anode right/cathode left	20	F4	F3	2 mA	–	
	Sham tDCS with the same electrode placement	20	F4	F3	–	The stimulation automatically turned off after 30 s	
Khedr et al., 2014	One type o bilateral stimulation of anodal tDCS, over the left DLPFC (Reference electrode over the contralateral arm)—10 sessions (5 sessions/week)	25	6 cm anterior to the left (M I)	–	2 mA	No sham condition	NS
Lapenta et al., 2014	Two types of bilateral stimulation of DLPFC (1 week ii)—1 session	–	–	–	–	–	NS
	Active tDCS, anode right/cathode left	20	F4	F3	2 mA		
	Sham tDCS with the same electrode placement	20	F4	F3	–	The stimulation automatically turned off after 30 s	
Jauch-Chara et al., 2014	Two types of bilateral stimulation of DLPFC (2 weeks ii)—8 daily sessions						All sensations were transient and ranged from mild to moderate: skin redness (*n* = 9), tingling (*n* = 4), itching (*n* = 7), and feelings of skin burning (*n* = 2).
	Active tDCS, anode right/cathode left	20	5 cm method	Over the left forehead	1mA		
	Sham tDCS	20	NS	NS	–	NS	

*DLPFC, Dorso Lateral Prefrontal Cortex; F3, 10–20 EEG system; F4, 10-20 EEG system; ii, intersession interval; M I, primary motor cortex; NS, Not Specified; tDCS, transcranial Direct Current Stimulation*.

## Results

The initial search identified 402 independent articles. Seven articles met the criteria for inclusion. Food craving, in different clinical conditions was the only symptom to be tested. To the best of our knowledge, we found that tDCS has not yet been tested for the following BAs: compulsive buying/shopping, pathological gambling, gambling disorder, Internet addition, video game addiction, sex addiction and sports addiction.

### Efficacy of tDCS in behavioral addictions

The main characteristics of the studies are summarized in Table 1.

Six out of the seven published studies (Fregni et al., 2008b; Goldman et al., 2011; Montenegro et al., 2012; Jauch-Chara et al., 2014; Kekic et al., 2014; Lapenta et al., 2014) have demonstrated the efficacy of tDCS applied to the DLPFC in reducing food craving. Khedr et al. reported an improvement in anorexic conducts (Khedr et al., 2014). Two of these studies were led by the same team (Fregni et al., 2008b; Lapenta et al., 2014) with the same design. All studies but one (Khedr et al., 2014) were blinded, randomized, and controlled. The used sample sizes vary between 7 (Khedr et al., 2014) and 23 (Fregni et al., 2008b) subjects. The participants were majoritarily women aged less than 30 years old on average, in good health, and with frequent food cravings. Only one study included overweight patients (Montenegro et al., 2012), and only one included anorexic patients (Khedr et al., 2014). In all studies but two (Montenegro et al., 2012; Khedr et al., 2014), the craving was induced visually, either with images, or with real food. The craving was induced through visual stimuli before and after stimulation in four of the seven studies (Fregni et al., 2008b; Goldman et al., 2011; Kekic et al., 2014; Lapenta et al., 2014). One of the studies repeated the induction after half of the stimulation time (Goldman et al., 2011). Three studies used exposure to real, high-calorie food, combined with one or two short movies showing high-calorie foods (Fregni et al., 2008b; Kekic et al., 2014; Lapenta et al., 2014). One study used pictures of high calorie food items to induce craving (Goldman et al., 2011). Both types of craving induction were reported to lead to increased craving. The level of food craving was usually measured before and after stimulation by means of visual analog scales (VAS) with the exception of one study which did not assess craving at all (Khedr et al., 2014). Some studies used additional measures such as eye tracking (Fregni et al., 2008b; Lapenta et al., 2014) or the Food Craving Questionnaire-State (Kekic et al., 2014). Five studies assessed actual food intake after stimulation using a bogus taste test (Fregni et al., 2008b; Goldman et al., 2011; Jauch-Chara et al., 2014; Kekic et al., 2014; Lapenta et al., 2014).

Further assessment methods were also used, either clinical with specific impulsivity scales (Kekic et al., 2014), or physiological like visual attention, measured by eye tracking (Fregni et al., 2008b), salivary cortisol levels (Kekic et al., 2014) or event-related potentials (Lapenta et al., 2014).

### tDCS technical procedures

The results are summarized in Table 2.

Most studies tested the effect of just one active tDCS session vs. a sham tDCS session (20 min, 2 mA) on food craving. No cortical target other than the DLPFC was tested. Electrodes were most often placed with the anode on the right and the cathode on the left, respectively on F4 and F3 according to the International 10–20 System. Three teams placed them the other way around (cathode on the right and anode on the left) (Fregni et al., 2008b; Montenegro et al., 2012; Khedr et al., 2014). Montenegro and colleagues had two comparing arms (active tDCS and placebo) (Montenegro et al., 2012), whereas Fregni et al. had three comparing arms (anode/right and cathode/left; anode/left and cathode/right; placebo) (Fregni et al., 2008b). The interval between two sessions (active and placebo) ranges from 48 h to a week, to avoid a carry-over effect. The placebo method was described more or less precisely in all studies but one (Jauch-Chara et al., 2014). Tolerance and side effects were reported in 50% of studies (Fregni et al., 2008b; Jauch-Chara et al., 2014; Kekic et al., 2014).

## Discussion

### General instructions

The initial works on tDCS in BAs are recent, and started around the same period (Fregni et al., 2008b) as studies on tDCS in SUDs (Boggio et al., 2008; Fregni et al., 2008a). However, they have not generated the same intererest overtime, so that the application of tDCS in SUDs has been much more investigated than tDCS in BAs. Works on tDCS in BAs were first and only interested in eating behavior, based on the model of rTMS, which is another NIBS which efficacy in BAs was first tested in eating disorders (Grall-Bronnec and Sauvaget, 2014). Whether through rTMS or tDCS, no study has been conducted to this day on other BAs (pathological gambling, sexual addiction, sports addiction, Internet addiction, compulsive shopping) (Grall-Bronnec and Sauvaget, 2014). Furthermore, although tDCS is a more manageable and less expensive means than rTMS (Brunoni et al., 2013), we observe that fewer studies are conducted with tDCS in the field of BAs and SUD compared to rTMS (Grall-Bronnec and Sauvaget, 2014). The later development of tDCS could explain the smaller number of studies.

tDCS is found effective in reducing craving in BAs in controlled studies comparing stimulation vs. placebo, until now for food craving. These results point in the same direction as those of tDCS for SUDs, which have been consolidated by a recent meta-analysis arguing that applying NIBS to the DLPFC decreases craving levels in substance dependence (Jansen et al., 2013), without any significant difference between rTMS and tDCS. However, the efficacy of tDCS must be discussed in light of methodological and technical considerations. All possible biases have been discussed (see Table 3).

**Table 3:** **Main sources of bias in the studies of tDCS in behavioral addictions**.

**SELECTION BIAS**
Method of recruiting subjects (healthy participant, non-healthy participant, with or without treatment participants).
Duration and severity of the addiction or related disorder. Stage of treatment prior to tDCS (detoxification or continuation of substance use).
**OBSERVATION BIAS**
Over or underestimating the intensity of craving.
Placebo effect of tDCS itself.
Placebo effect of therapeutic trials carried out in the field of addiction and related disorders.
Order of the placebo session and active session in a crossover study.
Insufficient number of pulses and number of sessions.
Attrition bias (drop out).
**CONFOUNDING BIAS**
Sociodemographic characteristics: age, gender, ethnicity.
Hormonal status.
Volume of gray matter.
Psychiatric and somatic comorbidities.
Handedness.
Psychotropic treatments (in particular, continuation of anti-craving drugs during the trial).
Duration of the session, which may overlap with the duration required for the craving to subside naturally.
Cumulative and persistent effects of tDCS when the interval between two sessions is very short.
Sample size.
Ability of the treatment-seeking participants to use relapse prevention techniques during cue-induced craving procedure.

*All these biases are discussed in Sections Methodological Issues and Technical Issues*.

### Methodological issues

#### Characteristics of the participants

##### Health status of the participants

Patient inclusion criteria can be relatively confusing: indeed, most participants are defined as “healthy” subjects, whereas the study aims at investigating the effect of brain stimulation on food craving, which is a clearly defined disorder from a psychopathological standpoint. The frequency of food craving is relatively low in tDCS studies. It varies, depending on the studies, from 1/day (Kekic et al., 2014) to 3/day (Fregni et al., 2008b; Goldman et al., 2011; Lapenta et al., 2014). Moreover, most patients included in studies on tDCS have a normal weight, apart from one study in obese patients (Montenegro et al., 2012) and another in patients suffering from anorexia nervosa (Khedr et al., 2014). Only Jauch-Chara et al.'s study can be considered as conducted in a physiological condition, since the included subjects had a normal body mass index and no daily food craving (Jauch-Chara et al., 2014). In fact, the studies were more interested in the process of food craving than in full-syndrome eating disorders. Patient morphology could be an important criterion to take into account in configurating tDCS. This precise point is developed in the “Technical Issues” Section.

##### Age

Participants are rather young (< 40 years old). The age of the studied population is important to interpret results since the clinical expression of craving is likely to evolve with age. Age is also a factor in the variation of cortical excitability (Feil and Zangen, 2010; Clark and Taylor, 2011).

##### Gender

Apart from Jauch-Chara et al.'s study (2014) in which all subjects are male, the other studies mainly included women, either on purpose (Kekic et al., 2014; Lapenta et al., 2014) or because they were predominant (Fregni et al., 2008b; Goldman et al., 2011; Montenegro et al., 2012; Khedr et al., 2014). A higher prevalence of food craving in women than in men explains the sex-ratio imbalance between patients included in these studies (Mitchison and Hay, 2014). Moreover, the fluctuation of eating behavior throughout the menstrual cycle could affect the result of the studies (Lester et al., 2003). The sexual hormonal variations could also affect the functional cerebral asymmetries (Neufang et al., 2009). The right hemispheric predominance in spatial attention, which seems linked to gender, would disappear under the effect of left anodal tDCS (de Tommaso et al., 2014). Finally, a recent study showed that electric transmission of tDCS is different between men and women, mainly for bone density reasons (Russell et al., 2014).

##### Handedness

None of the studies analyzed in this review evoked the importance of this parameter in the interpretation of results, conversely to other NIBS works, whether on rTMS (Van den Eynde et al., 2012) or tDCS (Kasuga et al., 2015). Yet, the effects of tDCS could differ according to the handedness of stimulated subjects (Kasuga et al., 2015). The problem of hemispheric dominance remains complex, since the left hemisphere could be the dominant hemisphere in 95–99% of right-handed subjects, and in 70% of left-handed subjects (Corballis, 2014). Moreover, laterality has clinical relevance since left-handers are more at risk of developing addictive disorders (Sperling et al., 2000). Evaluating the hemispheric dominance thanks to a specific questionnaire focused on laterality (Oldfield, 1971) in patients included in studies involving NIBS would allow gathering new data on brain functioning.

##### Main exclusion criteria

The exclusion criteria are generally mentioned and detailed. Although contra-indications are usually exclusion criteria, in accordance with the literature (Brunoni et al., 2011), the psychiatric and somatic comorbidities can be confounding factors in evaluating the efficacy of tDCS. For example, in eating disorders, the conjoint improvement of depressive symptoms and obsessive-compulsive symptoms on the one hand, and of binge-eating and purging conducts on the other hand, could simply be due to a common physiopathological process rather than to a specific effect of tDCS on the addictive symptoms only (Khedr et al., 2014; Shiozawa et al., 2014). Finally, the use of medication, particularly psychotropic and anticraving drugs, should be considered, as they could interfere with the assessment of craving, but also with the efficacy of tDCS, through their action on cortical excitability. Selective serotonin reuptake inhibitors could indeed aid tDCS response (Nitsche et al., 2009).

##### Sample size

Among all examined studies, the sample size is always very small, ranging between 7 (Khedr et al., 2014) and 23 subjects (Fregni et al., 2008b). This is probably why all of them, except for two studies (Montenegro et al., 2012; Khedr et al., 2014), adopted a crossover design, to increase the statistical power of their work.

#### Cortical excitability

tDCS modifies cortical excitability (Nitsche et al., 2008). The efficacy of tDCS thus depends on numerous factors, which have an influence on cortical excitability, such as age, gender, hormonal status, anxiety levels, lack of sleep, and the use of psychotropic drugs (either legal or illegal). Cortical excitability would also vary according to ethnic origins (Yi et al., 2014). The results of the studies should thus be discussed according to these parameters.

#### Design

All studies but one (Khedr et al., 2014) were designed following the rules of RCTs, which facilitates comparisons. Although food craving is the main evaluation criterion, some authors have underlined the importance of considering other target symptoms such as impulsivity (Kekic et al., 2014). In their studies, patients with “more reflective choice behavior” are more sensitive to the anti-craving effects of the tDCS than patients with “more impulsive choice behavior.” These results show that the craving involves multiple dimensions that interact with each other, and that can also be modified by tDCS.

The craving induction procedure differs between studies. Although the induction medium (either real or virtual) is most of the time visual, this may vary. Addressing other sense organs like olfaction may increase the external validity of craving induction methods. Although craving is not necessarily related to physiological hunger, food intake before the experimental session may be an important interfering factor. Most studies tried to control this variable, by asking participants to refrain from food intake for a period of time before the session, which varied between 2 and 6 h depending on the study design (Fregni et al., 2008b; Goldman et al., 2011; Montenegro et al., 2012; Jauch-Chara et al., 2014; Lapenta et al., 2014). Some also used a 24 h dietary recall protocol to assess previous food intake (Goldman et al., 2011; Montenegro et al., 2012). One study controlled food intake only by a VAS on hunger (Kekic et al., 2014), and another study did not control this factor at all (Khedr et al., 2014).

### Technical issues

#### General considerations

The tDCS procedure is generally well-described and detailed, which allows for a better comparison between studies. Teams that consecutively conduct several studies tend to replicate the same protocol (Fregni et al., 2008b; Lapenta et al., 2014).

#### Stimulation site

Only the DLPFC drew the researchers' attention, most often in the following setting: anode on the right DLPFC (excitation) and cathode on the left DLPFC (inhibition). The results of Lapenta et al.'s team suggest that stimulating the DLPFC could facilitate the inhibitory response and modify the connections between the cortical and subcortical structures (Lapenta et al., 2014).

The positive results both in overeating with the “anode on the right DLPFC and cathode on the left DLPFC” scheme and in food restriction with the “anode on the left DLPFC and cathode on the right DLPFC” scheme argue in favor of a different hemispheric functioning in eating disorders. In the case of overeating and obesity, increasing the activity of the right DLPFC and inhibiting the left DLPFC would help reducing the induced food craving. This could decrease appetite and restore food control mechanisms, in line with the “right brain hypothesis for obesity” theory (Alonso-Alonso and Pascual-Leone, 2007; Alonso-Alonso, 2013). In the case of food restriction, the hypothesis of an imbalanced interhemispheric balance (with hyperactive right frontal regions) combined with anorexia nervosa (Hecht, 2010) has been partly confirmed by Khedr's work (Khedr et al., 2014). The possible predominance of the right hemisphere in the genesis of an eating disorder has also been evoked in a post-lesional context (Uher and Treasure, 2005). These results are consistent with the works on rTMS that put forward the respective roles of the right and left DLPFC in the control of craving. Whereas, the left DLPFC seems to have a role in the control of craving (Hayashi et al., 2013), the right DLPFC seems to play a part in the inhibitory control of emotional impulses (Pripfl et al., 2013).

However, the studies published to this day do not all have the same methodology, clinical populations or objectives. The results they put forward are still at a very preliminary stage and do not allow concluding on a hemispheric specialization in eating disorders and in BAs generally speaking. Besides switching electrodes between the two hemispheres to test the two conditions (excitatory or inhibitory) (McClelland et al., 2013), it would be interesting to stimulate other cortical regions, such as the parietal cortex, which is a cerebral region that seems to be involved in body image (Gaudio and Quattrocchi, 2012).

The choice of the stimulation site must also take into account the neural loops involved in the studied BA. It seems more pertinent to choose the stimulation site based on the neural loops involved in the studied behavioral addiction, rather than sticking to a given cortical region.

#### General design of the sessions and tDCS parameters

Apart from two studies (Jauch-Chara et al., 2014; Khedr et al., 2014), the included studies only tested the effects of one tDCS session. It can be assumed that repeating sessions could increase and sustain efficacy on craving and other eating disorder symptoms, as reported in studies on the treatment of depressive disorders (Shiozawa et al., 2014) and SUDs (Feil and Zangen, 2010; Tortella et al., 2015).

tDCS is generally described as easier to implement than rTMS. However, this should not overshadow the importance of some tDCS parameters, which might have an influence on the results (Horvath et al., 2014).

Indeed, beside the optimal position of the electrodes, current intensity, and stimulation duration, other parameters should be taken into account, such as hair thickness, sweat (Horvath et al., 2014), but also electrode size (Russell et al., 2014; Nasseri et al., 2015), with individual differences (Russell et al., 2013). The location of the reference electrode may also have an impact on tDCS effects (Nasseri et al., 2015). The electric current in tDCS is not relayed in the same way by the different anatomical structures it passes through (Shahid et al., 2014). For example, regarding adipose tissue, Truong et al. have showed that, based on the MRI analysis of five patients' adipose tissue, that tissue could influence the intensity of tDCS electric current in the brain (Truong et al., 2013). Furthermore, patients suffering from eating disorders could present changes in gray matter and its thickness (Frank et al., 2013). Consequently, the transmission of tDCS electric current could be altered, and the results could differ from the intended target.

#### Sham procedure

The placebo conditions, as described more or less precisely in all studies are similar, and follow a validated method (Gandiga et al., 2006). The placebo tDCS method seems more reliable and easier to implement than the rTMS placebo method, thus limiting the interpretation biases (Grall-Bronnec and Sauvaget, 2014). However, some authors have evidenced that sham tDCS was not as reliable as it seemed (Horvath et al., 2014).

#### Safety and tolerance

When reported (Fregni et al., 2008; Jauch-Chara et al., 2014), the side effects were similar to those found in literature (Brunoni et al., 2011). Indeed, tDCS is generally known as a safe technique with mild and transient adverse effects (Brunoni et al., 2011). Even though seizures induced by tDCS are very rare (Ekici, 2015), subjects suffering from substance use addiction present an increased risk of seizures, especially during the alcohol or benzodiazepine withdrawal periods (Leach et al., 2012). The tDCS techniques could be tolerated better by patients suffering from BAs, as they are less likely to have seizures than patients with a SUD.

## Conclusion and future perspectives

The application field of tDCS in BAs is for now restricted to the study of food craving, mainly in so-called “sane” participants, i.e., who do not fulfill the diagnostic criteria of characterized eating disorders. These studies show that stimulating the right DLPFC and inhibiting the left DLPFC reduces the induced food craving.

Therefore, there is a clinical interest in having a symptomatic treatment of craving, by considering tDCS as a complementary therapy to the standard treatment of eating disorder. On a neuroscientific level, tDCS could reduce inter-hemispheric imbalance, since data report overactivity in the frontal area of the right hemisphere in anorexia nervosa, as ventured by Hecht in 2010 (Hecht, 2010), and recently comforted by Khedr et al.'s work (2014). Also, other therapeutic effects could be observed with tDCS, especially on food restriction (Jauch-Chara et al., 2014).

The rationale of expanding tDCS work to other behavioral addictions is justified by its positive effect in sane subjects on their decision-making process (improved) and on their risk-taking (reduced) (Fecteau et al., 2007a,b), both strongly linked to addictive issues. Indeed, the recent works on tDCS in the field of cognition and impulsivity (Feil and Zangen, 2010; Elmasry et al., 2015) demonstrate promising therapeutic prospects for tDCS.

The tDCS techniques offer many undeniable advantages in treating BAs: they are non-invasive, well-tolerated, implemented through a portable, and compact device, and relatively cheap compared to other techniques (such as rTMS). Thus, tDCS could be implemented in outpatient structures specialized in addictions.

Several research avenues must be explored, in line with the research conducted with rTMS. The effects of tDCS in other BAs could be explored, like pathological gambling, sports addiction, sexual addictions, or video games. It would also be particularly interesting to evaluate the effects of tDCS in the longer term, whether on craving or on other BA symptoms, such as maintained abstinence.

Finally, combining neuroimaging and electrophysiology studies (Val-Laillet et al., 2015; Wolz et al., 2015) to tDCS studies should be considered, to understand better the pathophysiological mechanisms involved in BAs, and allow for a better identification of targets and stimulation parameters.

In summary, the main goals of tDCS application in BAs are all at once therapeutic, by modulating craving, impulsivity, executive functions and physiopathological, by enhancing the knowledge of neurophysiological basis of BAs.

### Conflict of interest statement

The authors declare that the research was conducted in the absence of any commercial or financial relationships that could be construed as a potential conflict of interest.
